# p38 MAPK regulates the expression of ether à go-go potassium channel in human osteosarcoma cells

**DOI:** 10.2478/v10019-012-0043-x

**Published:** 2013-02-01

**Authors:** Xinyu Wu, Daixing Zhong, Bin Lin, Wenliang Zhai, Zhenqi Ding, Jin Wu

**Affiliations:** 1 Department of Neurology, the Affiliated Southeast Hospital of Xiamen University, Zhangzhou, China; 2 Department of Thoracic Surgery, the Affiliated Tangdu Hospital of Fourth Military Medical University, Xi’an, China; 3 Department of Orthopaedics, the Affiliated Southeast Hospital of Xiamen University, Zhangzhou, China

**Keywords:** Ether à go-go, cell proliferation, MAPK pathway, p53, osteosarcoma

## Abstract

**Background:**

The ether à go-go (Eag) channel has been shown to be overexpressed in a variety of cancers. However, the expression and function of Eag in osteosarcoma are poorly understood. In addition, the molecular mechanisms responsible for Eag overexpression in cancer cells remain unclear.

**Methods:**

The expression of Eag in human osteosarcoma cell line MG-63 was detected by reverse transcription polymerase chain reaction (RT-PCR) and Western blot analysis. The effect of Eag inhibition on MG-63 cell proliferation was assessed *in vitro*. The effect of short hairpin RNA (shRNA) mediated knockdown of Eag on osteosarcoma growth was evaluated in xenograft model *in vivo*. The activation of mitogen-activated protein kinase (MAPK) pathway and p53 in MG-63 cells was detected by Western blot analysis.

**Results:**

Eag was overexpressed in MG-63 cells. Imipramine or Eag shRNA significantly suppressed the proliferation of MG-63 cells *in vitro* and *in vivo*. MG-63 cell proliferation was specifically inhibited by p38 MAPK inhibitor SB203580 or small interference RNA (siRNA). The inhibition of p38 MAPK activation by SB203580 or siRNA reduced Eag protein level but increased p53 protein level. Moreover, the activation of p53 by nutlin-3 induced cell growth arrest in MG-63 cells and reduced Eag protein level, while the inactivation of p53 by pifithrin-alpha (PFT-α) promoted MG-63 cell growth and increased Eag protein expression.

**Conclusions:**

Eag channel functions as an oncogene to promote the proliferation of human osteosarocma cells. Furthermore, the high expression of Eag in osteosarcoma cells is regulated by p38 MAPK/p53 pathway.

## Introduction

Voltage-gated potassium channels (Kv) play a vital role in the function of diverse cell types. Ether à go-go (Eag) channels are a unique group of Kv channels due to their close relation to tumor growth, progression and metastasis. Eag channels were originally cloned from *Drosophila melanogaster*^1^ and consist of three subfamilies: EAG, ERG (the eag-related gene) and ELK (the eag-like gene).^2^ The physiological expression of Eag is restricted to the central nervous system and placenta^3^, but Eag is aberrantly expressed in several tumor cell lines and more than 75% of the primary solid tumors.^4^,^5^ In addition, Eag appears to induce tumor angiogenesis by the induction of functional increase of hypoxia inducible factor-1 (HIF-1) and the secretion of vascular endothelial growth factor (VGEF) upon hypoxia^6^ and participates in the acquisition of malignant phenotypes in lung tumor cell.^7^ Moreover, siRNA targeting of Eag^4^,^8^ or non-specific blockers^9^ have been shown to suppress the proliferation of tumor cells. Later studies confirmed that aberrant expression of Eag in tumors is associated with a poor prognosis.^10^,^11^ Taken together, these studies reveal the oncogenic potential of Eag.^12^,^13^

Osteosarcoma is the most common primary bone tumor,^14^ which is characterized by a high incidence among youngsters and the potential of lung metastasis.^15^,^16^ Despite the recent development of neoadjuvant chemotherapy and advances in surgical techniques, the overall relapse-free survival rate of osteosarcoma over 5 years remains approximately 65%.^17^ Moreover, 30% of osteosarcoma patients die of lung metastases when presenting with metastases at the time of diagnosis.^18^ Therefore, there is an urgent need to develop new treatment methods for osteosarcoma in the clinic.^19^

Although a clear relationship between Eag and tumour progression has been established, it is unclear whether Eag plays a similar oncogenic role in osteosarcoma. This study aimed to investigate the expression and function of Eag in human osteosarcoma. In addition, we explored the signaling mechanisms that contribute to Eag overexpression in osteosarcoma cells. In this study we employed loss of function of approach to inhibit Eag expression or activity and found the consequent inhibition of the proliferation of human osteosarcoma cells *in vitro* and *in vivo*. Furthermore, our data suggest that Eag expression is regulated by p38 MAPK/p53 pathway.

## Materials and methods

### Cell culture

Human osteosarcoma cell line MG-63, human osteoblastic cell line hFOB 1.19, human breast cancer cell line MDA-MB435S and human embryonic kidney cell line 293 (HEK293) were purchased from the American Type Culture Collection. MG-63, MDA-MB435S and HEK293 cells were cultured in RPMI-1640 medium supplemented with 10% (v/v) fetal bovine serum (FBS) (Gibco, Rockville, MD, USA), 100 U/ml penicillin, and 100 μg/ml streptomycin in a humidified atmosphere of 5% CO_2_ in the air at 37°C. hFOB 1.19 cells were cultured in ham’s F12/ Dulbecco’s modified Eagle medium (DMEM) (Gibco) supplemented with 10% (v/v) FBS, 100 U/ ml penicillin, and 100 μg/ml streptomycin in a humidified atmosphere with 5% CO_2_ at 33.5°C. All cells were subcultured every 3–4 days.

### Drugs and siRNAs

Imipramine was purchased from Sigma (St. Louis, MO, USA), dissolved in distilled water as 1 mM stock solution and stored at −20°C. The p38 MAPK inhibitor SB203580 was from Calbiochem (Darmstadt, Germany), dissolved in dimethyl sulfoxide (DMSO) as 10 mM stock solution and stored at −20°C. The c-jun N-terminal kinase (JNK) MAPK inhibitor CEP11004 was from Sigma, dissolved in DMSO as 4 mM stock solution, and stored at −20°C. The extracellular-regulated kinase 1/2 (ERK1/2) MAPK inhibitor PD98059 was obtained from Santa Cruz Biotechnology (CA, USA), dissolved in DMSO as 10 mM stock solution, and stored at −20 °C. p38 MAPK siRNA (#6564), JNK MAPK siRNA (#6232), ERK1/2 MAPK siRNA (#6560) and control siRNA (#6568) were obtained from Cell Signaling Technology® (Danvers, MA) and stored at −20°C. Nutlin-3 (p53 activator) and PFT-α (p53 inhibitor) were purchased from Sigma. Stock solution of Nutlin-3 (20 mg/ml) was prepared in DMSO and stored at −20°C. PFT-α was protected from light and stored under inert gas.

### Preparation of adenoviral shRNA vectors

The oligonucleotides targeting human Eag was designed and selected as the template: AGC CAT CTT GGT CCC TTA TAA, which shared no homology with other coding sequences in human by BLAST analysis. A ring sequence of 9 base pairs (TTC AAG ACG) existed between the sense and antisense strands. The shRNA was synthesis by Sangon Biotech (Shanghai, China). Plasmid pGeneSil-1, which was purchased from GeneSil Biotechnology (Wuhan, China), contained the human U6 promoter inserted between the BamHI and the HindIII sites. The shRNA-expressing cassette was subcloned into pAdTrack vector between the HindIII and the XbaI sites.^20^ The recombinant plasmid was linearized by digestion with restriction endonuclease, and subsequently cotransformed into *E. coli* BJ5183 cells with an adenoviral backbone plasmid, pAdEasy-1. Recombinant plasmids were selected for kanamycin resistance, and transduced into the HEK293 cells. A recombinant adenovirus expressing shRNA against Eag (Ad5-Eag-shRNA) was generated. The recombinant adenovirus control (Ad5-Control-shRNA), which carries a CTA GGT GTT CTA GTC TGG ACT and did not target any known human genes, was generated as control. All viruses were propagated and purified on a CsCl gradient using standard methods. The viruses were titered for viral particles using standard methods based on spectrophotometry at 260 nm. Functional titer (plaque forming units) was determined with a plaque assay on HEK293 cells according to the method developed by Quantum Biotechnology.

### Adenovirus infection

MG-63 cells (1 × 10^5^) in serum-free RPMI-1640 were infected with Ad5-Eag-shRNA or Ad5-ControlshRNA at 5 MOI (multiplicity of infection, calculated as PFU/cell numbers) in a humidified atmosphere of 5% CO_2_ at 37°C. Virus-containing medium was removed 8 h later and replaced with fresh RPMI-1640 medium containing 10% (v/v) FBS. Cells were incubated for another 48 h.

### RT-PCR

The total RNA was isolated from the cultured cells by Trizol reagent (Invitrogen, Rockville, MD, USA). RNA purity and integrity was checked by running an aliquot on a denaturing 1% (w/v) agarose gel. cDNA was then synthesized from 1 μg of total RNA using 200 U reverse transcriptase (Takara, Tokyo, Japan), plus 200 μM dNTPs and 2.5 μM oligo-dT primer, in a 20 μL reaction volume, for 10 min at 30°C then 60 min at 42°C and finally at 80°C for 5 min. 1 μL of cDNA were then amplified by PCR in 25 μL reaction containing 2.5 U DNA polymerase and 200 μM dNTPs. Sequences of forward and backward primers, amplified fragment sizes, annealing temperatures were as follows: Eag, 5′-GCT TTT GAG AAC GTG GAT GAG-3′, 5′-CGA AGA TGG TGG CAT AGA GAA-3′, 475 bp, 56°C. β-actin, 5′-TCC ACC TTC CAG CAG ATG TG-3′, 5′-GCA TTT GCG GTG GAC GAT-3′, 75 bp, 54°C. Samples of PCR products were run on a 2% (w/v) agarose gel and the bands were visualized by ethidium bromide staining on a UV trans illuminator. Each experiment was repeated three times. Some of PCR products were sequenced to check the PCR specificity.

### Western blot analysis

5–6 × 10^7^ cells were collected and lysed in ice-cold lysis buffer containing 50 mmol/L Tris-Cl (pH 7.5), 150 mmol/L NaCl, 0.2 mmol/L EDTA, 1 mmol/L PMSF and 1% (v/v) Nonidet-P40 for 30 min. The lysates were centrifuged at 13,200 rpm for 10 min at 4°C and the supernatants were collected. 25 μg protein were resolved by a 12% SDS-PAGE and blotted on nitrocellulose membranes (Bio-Rad, Richmond, CA). Membranes were blocked with 10% (w/v) nonfat milk powder at room temperature for 1 h, and then incubated with antibodies to Eag (Alomone laboratories, Jerusalem, Israel), actin, phospho-ERK1/2, ERK1/2, phospho-JNK, JNK, phospho-p38 MAPK, p38 MAPK (Cell Signaling) and p53 (Abcam, Cambridge, MA) overnight, followed by incubation with horseradish peroxidaseconjugated goat anti-rabbit or anti-mouse secondary antibody (Santa Cruz Biotechnology). Then the membranes were developed with chemiluminescent detection kit (Zhongshan Biotechnology, Beijing, China) and exposed to X-ray films. Experiments were performed at least three times with representative data presented.

### Cell proliferation assay

The cell proliferation was analyzed by using Cell Counting Assay Kit-8 (CCK-8) (Dojindo Molecular Technologies, Gaithersburg, MD) according to the manufacturer’s protocol. In brief, 1 × 10^5^ cells were starved in serum-free medium for 12 h and then the cells were transduced. After 48 h, cells were harvested. Ten microliters of Cell Counting Assay Kit-8 solution was added to each well, the cells were incubated for another 1 h, and the absorbance (A) at 450 nm was measured by using spectrophotometer (Bio-Rad). Experiments were performed at least three times with representative data presented.

### Tumour model

Thymus-null BALB/c nude mice (female, age 6–8 weeks) were obtained from the Animal Center of Chinese Academy of Medical Sciences. All animal procedures were performed according to approved protocols and in accordance with recommendations for the proper use and care of laboratory animals. Osteosarcoma xenografts were established in nude mice according to a previous report.^21^ A total of 1.5 × 10^6^ MG-63 cells in 150 μL Phosphate-buffered Saline (PBS) were subcutaneously injected in the hind right leg. One week later, the tumors grew to visible size. The osteosarcomabearing mice were randomly divided into 3 groups (6 in each group). Group 1 received intra-tumor injections with Ad5-Eag-shRNA (10 MOI) every 2 days (6 injections totally). Group 2 received intratumor injections of Ad5-Control-shRNA (10 MOI) every 2 days (6 injections totally). Group 3 received normal saline injection as controls. Tumor volume (cm^3^) was determined based on the following formula: ab^2^/2 where a was the length and b was the width of the tumor.^21^

### Statistical analysis

All the data were presented as mean ± standard error of mean (SEM). Statistical significance was determined using t-test or analysis of variance (ANOVA) using the SPSS18.0 program. p < 0.05 was considered as statistically significant difference.

## Results

### Expression of Eag in osterosarcoma cells

**FIGURE 1.**
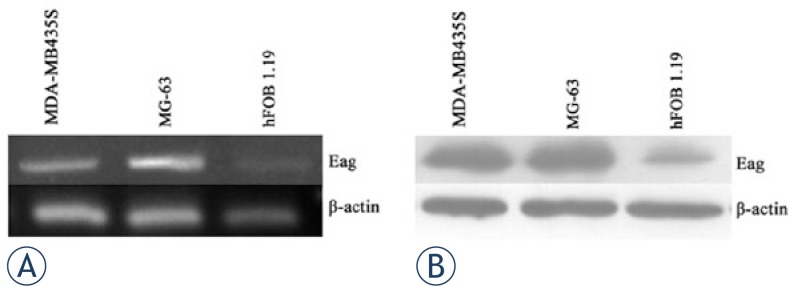
Expression of Eag is high in MG-63 cells. RT-PCR analysis of Eag mRNA level in three cell lines MDA-MB435S, MG-63 and hFOB 1.19 (A). Western blot analysis of Eag protein level in three cell lines MDA-MB435S, MG-63 and hFOB 1.19 (B). Shown were representative images from three independent experiments with similar results. MDA-MB435S and hFOB 1.19 cell lines served as positive and negative control, respectively. β-actin was used as a loading control.

RT-PCR analysis was performed to examine the expression of Eag mRNA in osteosarcoma cell line MG-63 and osteoblastic cell line hFOB 1.19. As a positive control, we used breast cancer cell line MDA-MB435S.^6^ Compared with hFOB 1.19 cells, the transcript of Eag was significantly increased in MG-63 cells (Figure 1A). Western blot analysis further confirmed the aberrant expression of Eag protein in MG-63 cells (Figure 1B).

### Eag silencing reduces the proliferation of osteosarcoma cells

**FIGURE 2.**
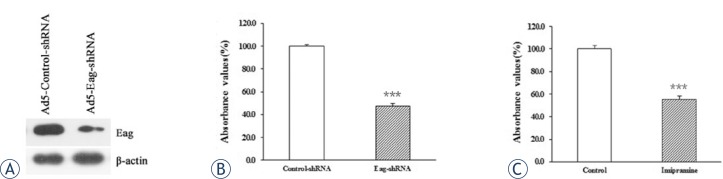
Inhibition of Eag expression or activity leads to reduced proliferation of MG-63 cells *in vitro*. The knockdown efficiency of Eag-shRNA in MG-63 cells was examined by Western blot analysis. β-actin was used as a loading control (A). CCK-8 assay showing that the proliferation of MG-63 cells was significantly reduced after transduction of Eag-shRNA (B) and treatment by 20 μM imipramine (C). Data were normalized as the value obtained for control or control-shRNA and presented as mean ± SEM (n = 8). ***p<0.001 vs. control.

To characterize the oncogenic role of Eag in osteosarcoma cells, we inhibited Eag expression by virus mediated silencing. Western blot analysis showed that Eag expression was suppressed in Ad5-Eag-shRNA infected MG-63 cells compared with Ad5-Control-shRNA infected cells (Figure 2A). Consequently, cell proliferation was inhibited by 56% (n = 8) in Ad5-Eag-shRNA infected MG-63 cells (Figure 2B). To further confirm the oncogenic role of Eag, we treated MG-63 cells with imipramine, a nonspecific blocker of Eag activity. The results showed that MG-63 cell proliferation was inhibited by 48% (n = 8) after the treatment with 20 μM imipramine (Figure 2C). Collectively, these results suggest that Eag promotes the proliferation of MG-63 cells *in vitro*.

### Eag silencing inhibits osteosarcoma growth *in vivo*

**FIGURE 3.**
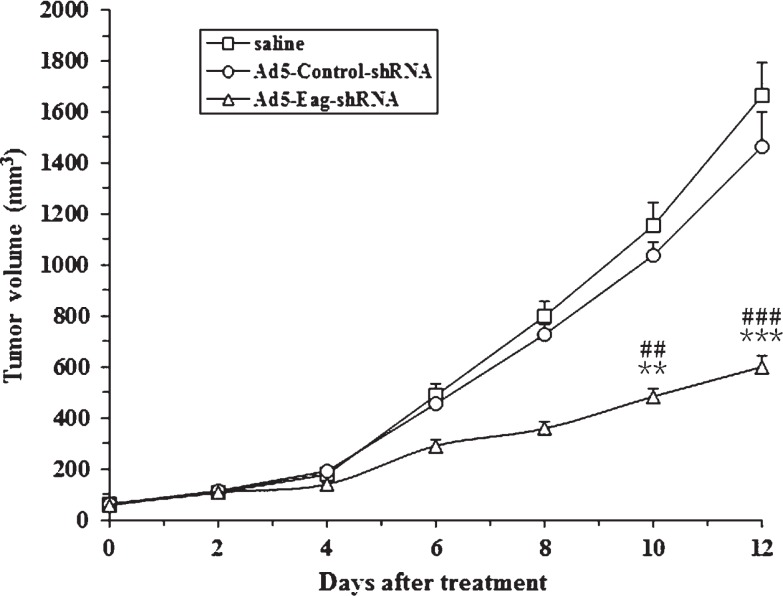
Eag silencing inhibits the growth of MG-63 cells in nude mice. Intra-tumor injection of Ad5-Eag-shRNA significantly reduced the size of MG-63 derived tumor implanted subcutaneously in nude mice during the 12-day follow-up period as compared with the saline control and the Ad5-Control-shRNA. **p<0.01; ***p<0.001, vs. the saline control; ^##^p<0. 01; ^###^p<0.001, vs. Ad5-Control-shRNA; (n = 6)

To investigate the in vivo role of Eag in osteosarcoma, we made a xenograft model of osteosarcoma using nude mice, and treated the xenografts by intra-tumor injection of Ad5-Eag-shRNA, Ad5-Control-shRNA or saline. The results showed that the tumor volume was significantly smaller in Ad5-Eag-shRNA injected animals compared to saline or Ad5-Control-shRNA injected animals at each evaluating time point (Figure 3). These *in vivo* data confirm our *in vitro* results and suggest the oncogenic role of Eag in osteosarcoma.

### p38 MAPK pathway is activated and promotes the proliferation of osteosarcoma cells

**FIGURE 4.**
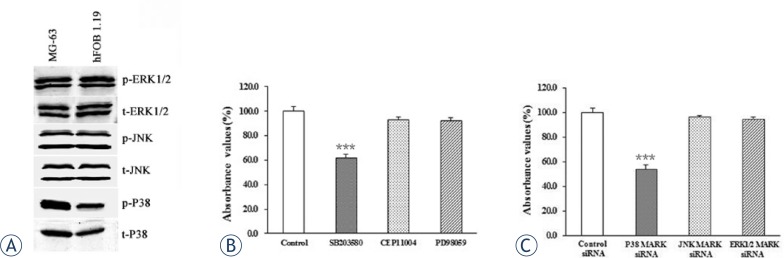
p38 MAPK pathway is activated and promotes the proliferation of MG-63 cells. Western blot analysis of the activation of MAPK pathways in MG-63 and hFOB 1.19 cells. Shown were representative blots from three independent experiments with similar results. Specifically, the phospho-p38 MAPK level was higher in MG-63 cells than in hFOB 1.19 cells. β-actin was used as a loading control (A). MG-63 cells were treated with different MAPK pathway inhibitors and cell proliferation was evaluated by CCK-8 assay (B). MG-63 cells were treated with different siRNAs against MAPK pathway components and cell proliferation was evaluated by CCK-8 assay (C). Data were normalized as the value obtained for control or control-siRNA and presented as mean ± SEM (n = 8); ***p<0.001 vs. control

To explore the molecular mechanism underlying the oncogenic role of Eag in osteosarcoma, we focused on MAPK pathway because Eag was shown to have a role in the activation of MAPK pathway which is frequently activated in a variety of tumors. 22–24 The activation of p38 MAPK, JNK MAPK and ERK1/2 MAPK were detected in MG-63 cells and hFOB 1.19 cells by Western blot analysis. As shown in Figure 4A, both total p38 MAPK and phospho-p38 MAPK levels were higher in MG-63 cells than hFOB 1.19 cells. Next we employed MAPK inhibitors and siRNA against MAPK to treat MG-63 cells. CCK-8 assay showed that the proliferation of MG-63 cells was significantly decreased by p38 MAPK inhibitor SB203580 and p38 MAPK siRNA, but not by JNK inhibitor CEP11004, JNK siRNA, ERK1/2 MAPK inhibitor PD98059, or ERK1/2 siRNA (Figure 4B,C). Taken together, these data indicate that high expression of Eag may induce the activation of p38 MAPK which then promotes the proliferation of MG-63 cells.

### p38 MAPK pathway modulates the expression of Eag in osteosarcoma cells

**FIGURE 5.**
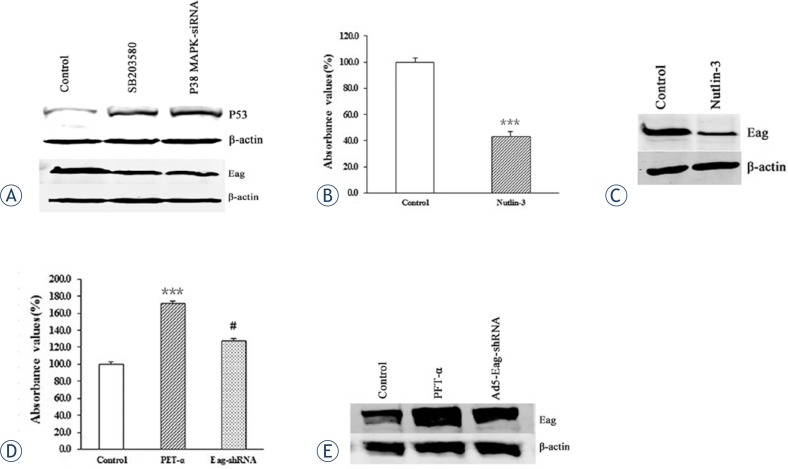
p38 MAPK pathway modulates the expression of Eag in MG-63 cells. Western blot analysis of p53 and Eag protein levels in MG-63 cells treated by SB203580 or p38 MAPK-siRNA (A). MG-63 cells were treated with nutlin-3 (1 μM) and cell proliferation was evaluated by CCK-8 assay (B). ***p < 0.001 vs. control. MG-63 cells were treated with nutlin-3 (1 μM) and Eag protein level was detected by Western blot analysis (C). MG-63 cells were treated with PFT-α (30 μM) alone or together with Ad5-Eag-shRNA and cell proliferation was evaluated by CCK-8 assay (D). MG-63 cells were treated with PFT-α (30 μM) alone or together with Ad5-Eag-shRNA and Eag protein level was detected by Western blot analysis (E). Data were normalized as the value obtained for control or control-siRNA and presented as mean ± SEM (n = 8). *** p<0.001 vs. control; # p<0.05 vs. PFT-α alone. Shown were representative blots from three independent experiments with similar results. β-actin was used as a loading control.

It has been proposed that p53 acts downstream of p38 MAPK pathway to modulate cell growth and apoptosis.^25^,^26^ Thus, we examined the expression level of p53 and Eag by Western blot analysis. A significant increase of p53 protein level and reduction of Eag protein level were observed in MG-63 cells treated by SB203580 or p38 MAPK siRNA (Figure 5A).

Next we treated MG-63 cells with p53 activator nutlin-3 and found that p53 activation induced cell growth arrest and reduced Eag protein level in MG-63 cells (Figure 5B,C). On the other hand, inactivation of p53 by PFT-α promoted cell growth and increased Eag protein expression, which were abrogated by Eag-shRNA (Figure 5D,E).

## Discussion

Imipramine, a tricyclic antidepressant, has been widely used in the psychiatric treatment for more than 50 years. The recent study showed that imipramine had a relative high affinity to Eag channels and permeate the membrane to inhibit the hEag1 current by selectively binding to open channels.^9^ The non-specific cytotoxic doses of imipramine is 50 μM.^27^ Thus, we used 20 μM imipramine in this study to avoid its cytotoxicity. We observed a significant reduction of proliferation in MG-63 cells treated by 20 μM imipramine for 2 days, which is consistent with previous reports.^4^,^28^,^29^ However, the side effects of imipramine limit its applicability in the cancer treatment.^6^ Compared with imipramine, siRNA is a more specific tool to investigate the role of Eag in cancer progression. siRNA mediated knockdown of Eag resulted in reduced proliferation of tumor cell lines without observable nonspecific responses.^8^ In our study, Eag-shRNA could effectively downregulate Eag expression in osteosarcoma cells with high specificity. In addition, Eag-shRNA inhibited the proliferation of osterosarcoma cells *in vitro* and *in vivo.*

Eag overexpression is known to be implicated in tumor progression. However, the intracellular signaling pathways downstream of Eag have not been fully characterized. MAPK pathway plays critical roles in the pathogenesis of various cancers^30^–^33^, including osteosarcoma.^34^,^35^ Results reported in our study demonstrated the correlation between high expression of Eag and the activation of p38 MAPK in MG-63 cells. It has been proposed recently that perinuclear localization of Eag could lead to the activation of MARP pathway resulting in increased cell proliferation.^36^ Therefore, it is possible that overexpression of Eag induces the activation of p38 MAPK which then promotes the proliferation of osteosarcoma cells because we showed that the inhibition of p38 MAPK/p53 pathway led to reduced MG-63 cell proliferation.

Interestingly, our results showed a significant increase of p53 protein level in MG-63 cells treated by p38 MAPK inhibitor or siRNA, suggesting that p53 acts downstream of p38 MAPK in MG-63 cells. Furthermore, p53 could modulate the expression of Eag in MG-63 cells as shown by the use of p53 activator and inhibitor. These data suggest that Eag and p38 MAPK may form a positive feedback loop to maintain the high expression of Eag in osteosarcoma cells. When Eag expression is high, it activates p38 MAPK, which in turn inactivates p53 and relieves the inhibition of Eag expression by p53. This is consistent with a recent report that Eag expression is negatively regulated by p53 through p53-miR34-E2F1 pathway in human neuroblastoma cells SHSY5Y.^37^

It is important to note that Eag contributes to tumor progression independently of its primary function as an ion channel. Previous study has shown that Eag interferes with oxygen homeostasis by increasing HIF-1 activity and VEGF secretion, thus promoting tumor vascularization.^6^ In this study our *in vitro* data using imipramine, a nonspecific blocker of Eag conduction activity, indicated that the proliferation of osteosarcoma cells depends on the K^+^ conducting activity of Eag. However, our *in vitro* and *in vivo* data using virus mediated Eag silencing to inhibit Eag expression suggest that other oncogenic role of Eag independent of its K^+^ conducting activity could not be excluded. Further studies are necessary to characterize the role of Eag in osteosarcoma development, especially angiogenesis and metastasis.

In summary, our results showed that Eag channel functions as an oncogene to promote the proliferation of human osteosarcoma cells. Furthermore, the high expression of Eag in osteosarcoma cells is regulated by p38 MAPK/p53 pathway. These findings suggest that the inhibition of p38 MAPK pathway or Eag overexpression is a promising approach for osteosarcoma therapy.
